# Novel picornavirus (family *Picornaviridae*) from freshwater fishes (*Perca fluviatilis, Sander lucioperca*, and *Ameiurus melas*) in Hungary

**DOI:** 10.1007/s00705-021-05167-y

**Published:** 2021-07-13

**Authors:** Renáta Hargitai, Péter Pankovics, Ákos Boros, Róbert Mátics, Eda Altan, Eric Delwart, Gábor Reuter

**Affiliations:** 1 Department of Medical Microbiology and Immunology, Medical School, University of Pécs, Szigeti út 12., 7624 Pecs, Hungary; 2 Hungarian Nature Research Society, Ajka, Hungary; 3 Vitalant Research Institute, San Francisco, CA USA; 4 University of California, San Francisco, CA USA

## Abstract

In this study, a novel picornavirus (perchPV/M9/2015/HUN, GenBank accession no. MW590713) was detected in eight (12.9%) out of 62 faecal samples collected from three (*Perca fluviatilis, Sander lucioperca*, and *Ameiurus melas*) out of 13 freshwater fish species tested and genetically characterized using viral metagenomics and RT-PCR methods. The complete genome of perchPV/M9/2015/HUN is 7,741 nt long, excluding the poly(A) tail, and has the genome organization 5’UTR^IRES-?/^P1(VP0-VP3-VP1)/P2(2A_1_^NPG↓P^-2A_2_^H-box/NC^-2B-2C)/P3(3A-3B^VPg^-3C^Pro^-3D^Pol^)/3’UTR-poly(A). The P1, 2C, and 3CD proteins had 41.4%, 38.1%, and 47.3% amino acid sequence identity to the corresponding proteins of Wenling lepidotrigla picornavirus (MG600079), eel picornavirus (NC_022332), and Wenling pleuronectiformes picornavirus (MG600098), respectively, as the closest relatives in the genus *Potamipivirus*. PerchPV/M9/2015/HUN represents a potential novel fish-origin species in an unassigned genus in the family *Picornaviridae*.

**Supplementary Information:**

The online version contains supplementary material available at 10.1007/s00705-021-05167-y.

## Introduction

Picornaviruses are small non-enveloped viruses with a positive-sense, single-stranded RNA genome, belonging to the family *Picornaviridae*. The family consists of 158 species grouped into 68 genera (https://www.picornaviridae.com/index.html). Not only is the known genetic diversity of the picornaviruses expanding rapidly but the number of known host species is also increasing. Picornaviruses have been identified in a wide range of vertebrates from fish to mammals, including humans. Most of the known picornaviruses were found in mammals and birds, but in recent years, novel picornaviruses have also been identified in lower vertebrates, including amphibians (newts) [16, 19], reptiles (tortoises) [8, 15], and fish. In the last group, six official fish-origin picornavirus genera – *Limnipivirus* from bluegill [4], carp [13], and minnow [18]; *Potamipivirus* from eel [9] and stickleback [12]; *Fipivirus* from sharpbelly, crossorhombus, jack mackerel, wrasse, and banjofish [20]; *Symapivirus* from triplecross lizardfish [20]; *Rajidapivirus* from sharpspine skate [20], and *Danipivirus* from zebrafish [1] – have been established and approved (https://talk.ictvonline.org/ictv-reports/ictv_online_report/positive-sense-rna-viruses/w/picornaviridae.https://www.picornaviridae.com/index.html), although there are further unassigned picornaviruses that have been discovered in different fish species [20].

Some picornaviruses are important viral pathogens, causing a wide range of diseases in humans and wild, domestic, and laboratory animals. However, little is known about the diversity, epidemiology, and disease-causing potential of fish picornaviruses [14].

In this study, we report the identification and complete genome characterization of a potentially novel picornavirus in freshwater fishes (*Perca fluviatilis, Sander lucioperca*, and *Ameiurus melas*) in Hungary.

## Materials and methods

A total of 62 faecal samples were collected directly from 13 different freshwater fish (Supplementary Table S1) living in natural and artificial open-air fishponds in the vicinity of Szarvas (Eastern Hungary) in the year 2015. The fish showed no clinical signs of disease during sample collection and were released immediately after sampling.

A specimen pool containing faecal samples from three European perch (M9, M13 and M15) were selected for viral metagenomics analysis. Briefly, 200 μl of PBS-diluted specimen was passed through a 0.45-μm sterile filter (Millipore) and centrifuged at 6,000 × *g* for 5 min. The filtrate was then treated with a mixture of DNases and RNases (Turbo DNase, Invitrogen; Baseline Zero DNase, Epicentre Biotechnologies; Benzonase Nuclease, Novagen; RNase A, Fermentas) at 37° C for 2 hours to digest unprotected nucleic acids [17]. Viral-particle-protected nucleic acids were extracted using a MagMAX Viral RNA Isolation Kit (Ambion, Austin, USA) with RNase inhibitor (RiboLock RNase Inhibitor, Fermentas) during the elution step. Sequence-independent random RT-PCR amplification [21] with 20 PCR cycles was used, and a viral cDNA library with 250 base-paired ends was constructed using a Nextera XT DNA Library Preparation Kit (Illumina). The library was sequenced on an Illumina MiSeq platform according to the manufacturer’s instructions. The metagenomic reads were trimmed, assembled *de novo* [7], and analyzed using an in-house pipeline [17]. Briefly, the singlets and assembled contigs greater than 250 bp were compared to the GenBank [6] protein database using BLASTx (version 2.2.7) [2], using an E-value cutoff of 0.01. Candidate viral hits were then compared to a non-viral non-redundant protein database to remove false positive viral hits. Virus-family-level categorization of all viral metagenomic sequences was based on the best BLASTx scores (E-value ≤ 10^-10^). Metagenomic raw sequence read data are available upon request.

A sequence-specific screening primer pair (PerchPV-screen1-F/R, Supplementary Table S2) was designed for the 3D region to identify the viral genome of the study strain from the specimen pool. The PCR thermocycler program consisted of 3 min at 95º C, 40 cycles for 30 s at 95º C, 20 s at 55º C, and 30 s at 72º C, and a final 3-min extension at 72º C using a C1000 Touch Thermal Cycler (Bio-Rad). In addition, different sets of specific primers (Supplementary Table S2) were designed based on the sequences of the metagenomics reads/contigs and the amplified PCR products for the verification of the metagenomics contig and to obtain the complete viral genome sequence, including the 5’ and 3’ ends, of the study strain (perchPV/M9/2015/HUN). All faecal samples were tested individually by the RT-PCR method, using the PerchPV-screen2-F/R (Supplementary Table S2) screening primer pair.

Sequence alignments of the P1, 2C, and 3CD regions were done using GeneDoc (version 2.7) and the MUSCLE web server based on the amino acid sequences of the studied fish picornavirus and representative picornaviruses available in the GenBank database. Phylogenetic analysis was performed using MEGA X (version 10.2.3), creating maximum-likelihood trees using the substitution model LG+F+G+I for P1 and 3CD analysis and LG+G+I for 2C, with 1000 bootstrap replicates as described previously [1]. Potential secondary RNA structures of the 5’ and 3’ UTRs of the analyzed picornavirus sequences were predicted using Mfold software [22].

The nucleotide (nt) and amino acid (aa) sequences of European perch picornavirus (perchPV/M9/2015/HUN) have been deposited in the GenBank database under the accession number MW590713.

## Results

A specimen pool containing three faecal samples from European perch was subjected to viral metagenomics analysis. After *de novo* assembly of the 16,612,456 sequence reads, 58,855 reads from this pool were found to show similarity (BLASTx cutoff E-value ≤ 10^-10^) to viral sequences. The sequences with more than 100 reads were from unclassified viruses (n = 13,542), as well as the families *Parvoviridae* (n = 15,209), *Dicistroviridae* (n = 12,081), *Reoviridae* (n = 5,360), *Picornaviridae* (n = 3,198), *Microviridae* (n = 2,689), *Circoviridae* (n = 1,825), *Tombusviridae* (n = 794), *Permutotetraviridae* (n = 643), *Phycodnaviridae* (n = 289), *Virgaviridae* (n = 170), and *Nanoviridae* (n = 133).

Sequence reads/contigs corresponding to family *Picornaviridae* were selected for further analysis. The longest picornavirus contig was 3,423 nucleotides (nt) long, and the encoded protein had only 40.2% aa sequence identity to the 2C/3C-3D region of eel picornavirus 1 strain F15/5 (NC_022332), a member of the species *Potamipivirus A* in the genus *Potamipivirus* [9]. A sequence-specific screening primer pair was designed based on the 3D region of the picornavirus sequence contig (Supplementary Table S2) to identify the picornavirus strain in the specimen pool. One of the three specimens from European perch was RT-PCR positive, and this sample (M9) was selected for further study.

**Fig. 1:**
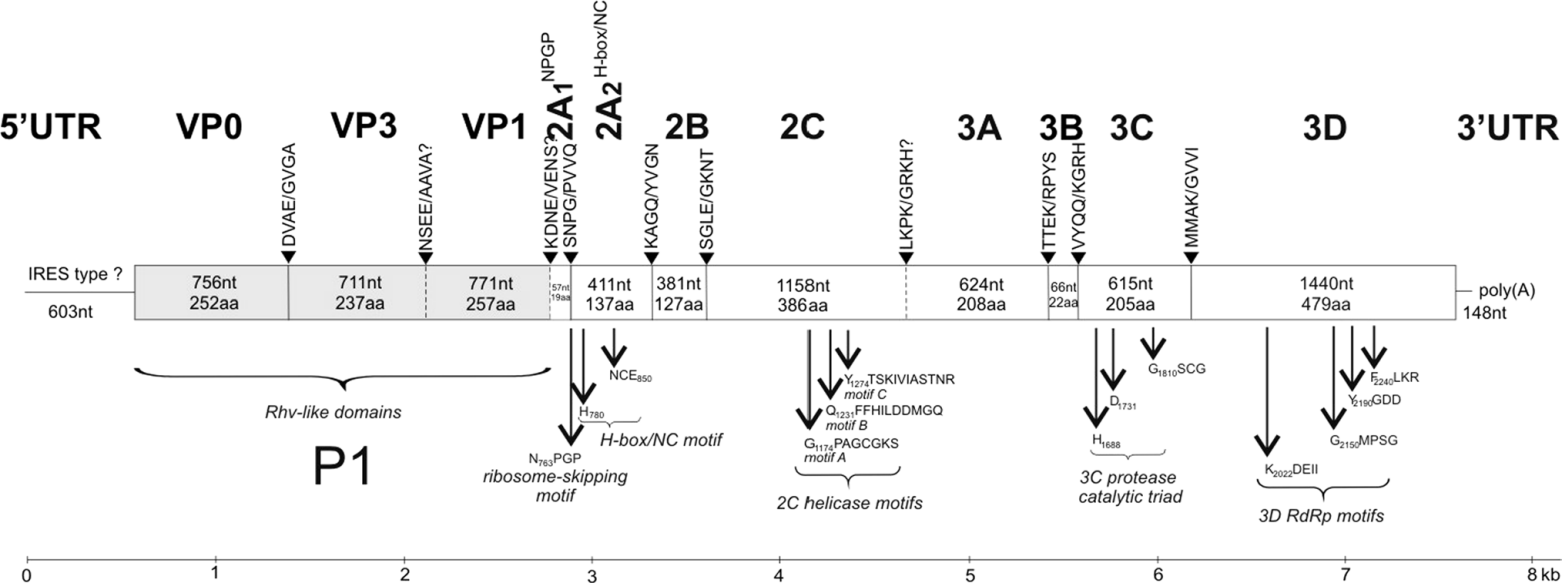
Schematic genome organization of perch picornavirus strain perchPV/M9/2015/HUN (GenBank accession no. MW590713). The genome map is drawn to scale. VP0, VP3, and VP1 represent viral structural proteins and are shown in grey. Nucleotide (upper number) and amino acid (lower number) lengths are indicated in each gene box. Conserved picornaviral amino acid motifs and predicted P4/P4’ cleavage sites are indicated above the genome map. Question marks and dotted lines indicate the uncertain borders of VP3/VP1, VP1/2A_1_, and 2C/3A. The IRES type of the 5’UTR is unknown (see Fig. 2). aa, amino acid; nt, nucleotide; RdRp, RNA-dependent RNA polymerase; UTR, untranslated region; VP, viral protein

The complete genome – including the complete coding regions – of European perch picornavirus (perchPV/M9/2015/HUN, MW590713) is 7,741 nt long excluding the poly(A) tail (Fig. 1). The genome organization is as follows: 5’UTR^IRES-?/^P1(VP0-VP3-VP1)/P2(2A_1_^NPG↓P^-2A_2_^H-box/NC^-2B-2C)/P3(3A-3B^VPg^-3C^Pro^-3D^Pol^)/3’UTR-poly(A). It has 44.4% G+C content and a nucleotide composition of 31.6% A, 24% U, 23.4% G, and 21% C. No nucleotide tandem repeats were found in the genome sequence.

**Fig. 2:**
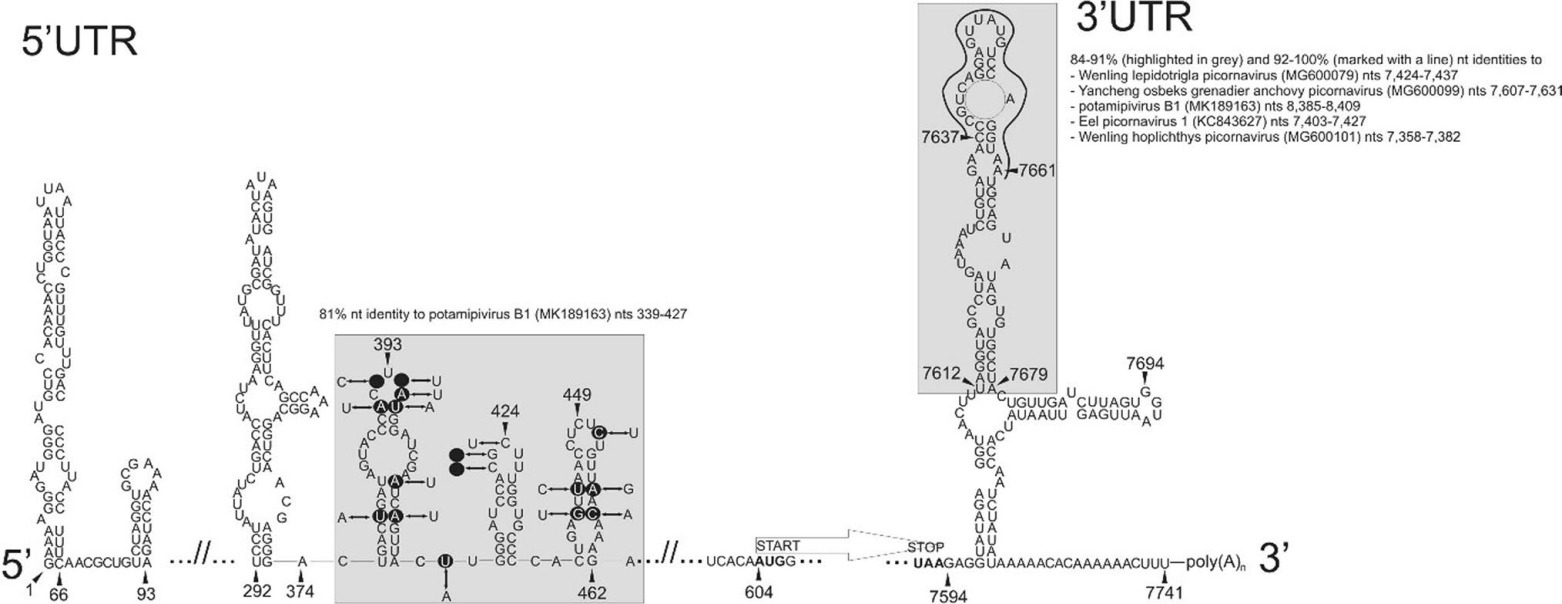
Predicted RNA secondary structure of the 5’UTR (left side; partial) and 3’UTR (right side; complete) of the genome sequence of perchPV/M9/2015/HUN. Significant nucleotide sequence similarity to other fish-origin picornaviruses, identified using BLASTn, are highlighted with a grey background. In the 5’UTR, the majority of the nucleotide mutations maintained their base pairing (and therefore the predicted secondary RNA structures) when perchPV/M9/2015/HUN and potamipivirus B1 (MK189163) were compared. In the 3’UTR, the stem-loop structure at the apex (indicated by a line) is highly conserved (92-100% identity) in perchPV/M9/2015/HUN and five other fish-origin picornavirus nucleotide sequences. The protein coding region extends from nucleotides 604 to 7593 in perchPV/M9/2015/HUN in a single open reading frame (ORF).

The European perch picornavirus 5’UTR is 603 nt long. The first in-frame AUG initiation codon is at nt position 604-606 in an optimal Kozak context (CACA**A**_**604**_**UG**G, start codon in bold). Using a GenBank BLASTn search, a unique, 90-nt-long region with partial genetic similarity was found in the 5’UTR region of potamipivirus B1 (species *Potamipivirus B*, MK189163) from stickleback [12]. This nucleotide region (between nt position 374 and 463 of the 5’UTR of European perch picornavirus) has 81% nt sequence identity to the corresponding non-coding region of potamipivirus B1 (Fig. 2). The secondary RNA structure of the 5’UTR including the IRES is unknown in potamipiviruses. Based on the 5’UTR sequences of European perch picornavirus and the above 5’UTR sequences of potamipivirus B1, the construction of a predicted RNA secondary structure model was partially successful (Fig. 2). The sequence alignment showed that the majority of the nucleotide mutations maintained their base pairing (and therefore the predicted secondary RNA structure) in the aligned region regardless of the Watson-Crick or wobble nature of the base pairing. However, none of the five known picornavirus IRES structures (types I-V) or the known conserved 5’UTR-IRES nt motifs [5] could be identified in the new sequence.

The predicted length of the coding region (ORF) is 6,990 nt, and it encodes a single 2,329-aa-long polyprotein (Fig. 1). The predicted capsid proteins (VP0, VP3, and VP1) had the highest aa sequence identity of 51.79% (coverage: 99%), 43.05% (coverage: 92%), and 38.79% (coverage: 88%) to the corresponding proteins of Wenling lepidotrigla picornavirus (MG600079), eel picornavirus strain F15/05 (NC_022332), and Wenling lepidotrigla picornavirus (MG600079), respectively, using GenBank BLASTp. The complete capsid polyprotein (P1) shows the highest sequence identity (41.4%; coverage: 96%) to the corresponding polyprotein of Wenling lepidotrigla picornavirus (MG600079). In the non-structural region, two potential 2A proteins were predicted: the 19-aa-long 2A_1_^NPG↓P^ and the 137-aa-long 2A_2_^H-box/NC^ (Fig. 1). The 2C possesses the GxxGxGKS (G_1174_PAGCGKS) motif for nucleotide triphosphate binding and the D_1237_DMGQ motif for putative helicase activity [11]. The 2C protein shows the highest aa sequence identity (38.1%, coverage: 94%) to the corresponding protein of eel picornavirus (NC_022332). The picornavirus 3C protease catalytic triad and well-conserved aa motifs of 3D (RNA-dependent RNA polymerase) are present in the study strain (Fig. 1) [3, 10]. The 3CD proteins show the highest aa sequence identity (47.3%, coverage: 99%) to the 3CD proteins of Wenling pleuronectiformes picornavirus (MG600098) from flatfish [20].

The 3’UTR is 148 nt long. Partial nucleotide sequence similarity (84-91% identity) was found between nt position 7,612 and 7,679 of the 3’UTR of European perch picornavirus to the 3’UTR regions of five picornaviruses, including Wenling lepidotrigla picornavirus (MG600079), Yancheng osbecks grenadier anchovy picornavirus (MG600099), potamipivirus A (MK189163), eel picornavirus (KC843627), and Wenling hoplichthys picornavirus (MG600101), using GenBank BLASTn search (Fig. 2). All of these picornaviruses were identified in different fish species. Especially high (92-100%) nt sequence identity was found within the region between nt position 7,637 and 7,661 of the 3’UTR of European perch picornavirus (Fig. 2). We used these six 3’UTR nt sequences to predict the RNA secondary structure of the 3’UTR (Fig. 2). We found that the majority of the nucleotide mutations maintained their base pairing (data not shown), and therefore, the predicted RNA secondary structure, especially that of the stem-loop at the apex is highly conserved in all of the sequences (Fig. 2). The 3’UTR has 64.2% A+U content.

**Fig. 3:**
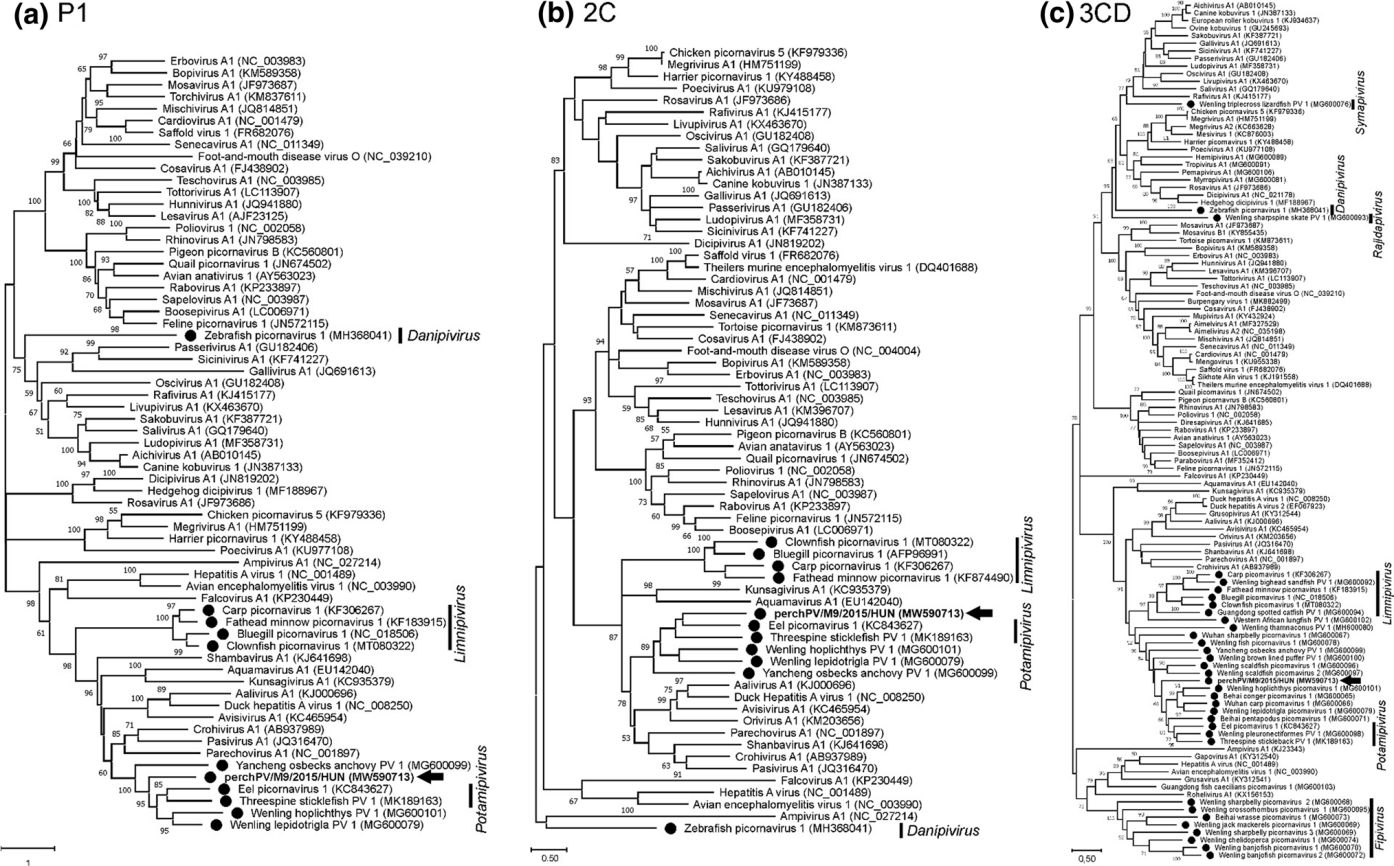
Phylogenetic analysis of the (a) P1, (b) 2C, and (c) 3CD proteins of prototype picornaviruses representing picornavirus genera and fish-origin picornaviruses, including the study strain perchPV/M9/2015/HUN (MW590713, bold letter and black arrows). Fish-origin picornaviruses are indicated by black dots. The names of the officially accepted picornavirus genera of fish origin are indicated. The 3CD tree includes all of the available fish-origin picornavirus 3CD protein sequences in the GenBank database. The P1 and 2C trees show only selected fish-origin picornavirus P1 and 2C protein sequences, mainly those most closely related to the study strain. Phylogenetic trees were constructed in MEGA X, using the maximum-likelihood method with the LG+F+G+I model for the P1 and 3CD regions and the LG+I+G model for the 2C region. Bootstrap values (based on 1000 replicates) for each node are given if >50%.

Phylogenetic analysis (Fig. 3) based on the amino acid sequences of the P1, 2C, and 3CD proteins showed that perchPV/M9/2015/HUN represents a distinct lineage among picornaviruses, but it is clustered together with the main group of known but presently unassigned fish picornaviruses.

The prevalence of the novel picornavirus genome was tested by RT-PCR in faecal samples from a total of 13 different freshwater fish species (Supplementary Table S1). A total of eight (12.9%) of the 62 faecal samples from three fish species were positive by RT-PCR for the study strain: three (37.5%) of eight, two (28.6%) of seven, and three (75%) of four specimens from European perch, zander, and black bullhead, respectively (Supplementary Table S1). Sequence analysis revealed that all of the sequences of 257-bp-long PCR products obtained using the PerchPV-screen2-F/R primers were 100% identical to each other.

## Discussion

Since the first confirmed report in 2014 [4], accumulating data have shown that fish are important vertebrate hosts of picornaviruses and therefore should not be neglected in picornaviral studies. Theoretically, and from an evolutionary point of view, lower vertebrates have been infected with a larger number of viruses and with genetically more diverse viruses than mammals. At present, six official fish-origin genera of picornaviruses (*Limnipivirus, Potamipivirus, Fipivirus*, *Symapivirus*, *Rajidapivirus*, and *Danipivirus*) have been established, but several picornaviruses identified in saltwater and freshwater fishes are waiting for their official taxonomy to be determined [20]. This study reports the complete genome characterization and molecular epidemiology of an additional novel picornavirus from freshwater fish. According to the current ICTV *Picornaviridae* Study Group taxonomy guidelines (http://www.picornastudygroup.com/definitions/genus_definition.htm), one of the criteria for creating a new picornavirus genus is significant divergence of the orthologous protein sequences: exceeding 66% for P1 and 64% for 2C, 3C, and 3D. Based on complete genome sequencing and comparative analysis, the strain reported here may represent a novel species and form a potential novel picornavirus genus in a populated monophyletic group of picornaviruses identified in fish.

Partial but significant nucleotide sequence similarity was identified in the 5’ and 3’ UTRs of perchPV/M9/2015/HUN and five other fish-origin picornaviruses. The RNA secondary structure of these UTRs is not known, but they could have a common origin. Based on their sequence similarity and presumed structural analogy, the partial 5’UTR and the complete 3’UTR secondary RNA structure could be predicted. While the detailed 5’UTR structure, especially the type of the internal ribosome entry site (IRES) remains unknown, the stem-loop structure in the apical part of the 3’UTR is highly conserved in these virus sequences.

PerchPV/M9/2015/HUN was identified in faecal specimens from different fish living in geographically separated open-air fish pond habitats. Whether this virus infects and replicates in specific tissues in different fish species or is able to cause symptomatic infections remains to be determined.

Investigation of the virome, including picornaviruses, of lower vertebrates contributes to our understanding of viral evolutionary relationships and allows the identification of possible disease-causing picornaviruses associated with wild, experimental, and farmed fish bred for human consumption.

## Supplementary Information

Below is the link to the electronic supplementary material.

Supplementary file1 (DOCX 20 kb)

Supplementary file2 (DOCX 19 kb)

Supplementary file3 (DOCX 15 kb)
