# Supplementary material for: Novel picornavirus (family Picornaviridae) from freshwater fishes (Perca fluviatilis, Sander lucioperca, and Ameiurus melas) in Hungary
Source: Arch Virol. 2021 Jul 13;166(9):2627–32. doi: 10.1007/s00705-021-05167-y (PMC8322000; doi:10.1007/s00705-021-05167-y)
Supplement: Supplementary file 1 — Supplementary file1 (DOCX 20 kb) [file 705_2021_5167_MOESM1_ESM.docx]

**Table S1.** RT-PCR detection of perch picornavirus from faecal specimens and distribution in different freshwater fish species.

| **Fish species** | **No of samples tested** | **No of samples positive (%)** |
| --- | --- | --- |
| European perch (*Perca fluviatilis*) | 8 | 3 (37.5%) |
| Zander (*Sander lucioperca*) | 7 | 2 (28.6%) |
| Black bullhead (*Ameiurus melas*) | 4 | 3 (75%) |
| Freshwater bream (*Abramis brama*) | 13 | 0 |
| Blue bream (*Ballerus ballerus*) | 10 | 0 |
| White bream (*Blicca bjoerkna*) | 5 | 0 |
| Roach (*Rutilus rutilus*) | 5 | 0 |
| Volga pikeperch (*Sander volgensis*) | 3 | 0 |
| Prussian carp (*Carassius auratus gibelio*) | 3 | 0 |
| White-eyed bream (*Abramis sapa*) | 1 | 0 |
| European carp (*Cyprinus carpio*) | 1 | 0 |
| Sabre carp (*Pelecus cultratus*) | 1 | 0 |
| Silver carp (*Hypophthalmichthys molitrix*) | 1 | 0 |
