# Supplementary material for: Novel picornavirus (family Picornaviridae) from freshwater fishes (Perca fluviatilis, Sander lucioperca, and Ameiurus melas) in Hungary
Source: Arch Virol. 2021 Jul 13;166(9):2627–32. doi: 10.1007/s00705-021-05167-y (PMC8322000; doi:10.1007/s00705-021-05167-y)
Supplement: Supplementary file 2 — Supplementary file2 (DOCX 19 kb) [file 705_2021_5167_MOESM2_ESM.docx]

**Table S2.** Primers used in this study for complete genome determination and specimen screening.

| Primer name | Primer sequences |
| --- | --- |
| PerchPV-121F | 5’- GAATGAGTACCGTATGAATGGA -3’ |
| PerchPV-985R | 5’- AAGAGGATCTCATCGCAGCAA -3’ |
| PerchPV-716F | 5’- CGTCTTCAGCCTCACAAGCA -3’ |
| PerchPV- 1822R | 5’- TGGAGGATTGGAGTCCAATGT -3’ |
| PerchPV-1792F | 5’- GCCATTCTGGACATTGGACT -3’ |
| PerchPV-2946R | 5’- ATAATGTTTATAGAGGCCACGAT -3’ |
| PerchPV- 3196F | 5’- GCTAGAGCACTAGCTGTCTTT -3’ |
| PerchPV- 4294R | 5’- GTCCTTGGTATCCATCCATGT -3’ |
| PerchPV-4391F | 5’- CAATGGCAGATCTCCCAGATA -3’ |
| PerchPV- 5584R | 5’- ATTTAGCCAAGTGTGCATATTCT -3’ |
| PerchPV-screen1-F (5660) | 5’- ACAAGCATTTCTTTGAGACAG -3’ |
| PerchPV-screen1-R (6386) | 5’- GCCTTGGTGTAGTGTTCCTT -3’ |
| PerchPV-screen2-F (7163) | 5’- TTGTGACATACGGVGATGATGTT -3’ |
| PerchPV-screen2-R (7419) | 5’- TCCATGGCACCATTGTATYTTTTG -3’ |
| PerchPV-523R-5’RACE | 5’- GGTGTCTGCATATGTTTGAGTT -3’ |
| PerchPV-610R-5’RACE | 5’- GTTCCATTGTGATATTTGTCGTT -3’ |
| PerchPV-6709F-3’RACE | 5’- TCAAGATGCATTGAAGCTTGT -3’ |
