# Supplementary material for: Novel picornavirus (family Picornaviridae) from freshwater fishes (Perca fluviatilis, Sander lucioperca, and Ameiurus melas) in Hungary
Source: Arch Virol. 2021 Jul 13;166(9):2627–32. doi: 10.1007/s00705-021-05167-y (PMC8322000; doi:10.1007/s00705-021-05167-y)
Supplement: Supplementary file 3 — Supplementary file3 (DOCX 15 kb) [file 705_2021_5167_MOESM3_ESM.docx]

*Complete genome sequence of perchPV/M9/2015/HUN (MW590713)

GAAAAGGGATGGGATGTCCACAAACCTGGTAATTAATTACCCGTTTGTTTGACCCCCTTACCTTTCAACGCTGTCTAGGTGCGAAAACCTAGAATCCCACTTTACCCAACCCCTTAGTTTGAATGAGTACCGTATGAATGGATTTAGACACATCTAAATGTCTTGCAACTGGTCTCGAACTGATAGGCTGTAGCCCCTGTCAAATCTACACATTTGGAAGGAATTAGGCGCTCTTGCTCGGATGATCCATAATTCATTGGACGAAGTGGTAACACTAGGCCTTGCTTGTTATCCTATTATCTGACCATCTAAGGTTTATGCGATATACTATAAGTGATCGGTTTGACTTCAGCCAAAGGCAGGTCAACGAGGGACTGACTGATAGTACCCACTATGGATCGAATCAGTTACTTGGGATCCACGCTTTGGTGCCCCACTGAGTTAACCTCTCTGTTAACAAAGACTCTCGAACTCTGCAACATCTTTCTTTAAAACTCTCAAAACTCAAACATATGCAGACACCAATTTTGTTATCAACTAGCGACTTATTAAATTCTCTGACATTTGAAATATTAAAGAAGTATTAAAACGACAAATATCACA**ATG**GAACTTTTCAAGAACGTTACACACAACTTAGCCAGCATTTTACAGGATCCTGCCAAGGAACAGGCCACCAATTCTTCAGACAGGGTTGGTGGGACATTGTCTATCAATGCG

TCTTCAGCCTCACAAGCAGTTGGAATCAGAAAGAGTGGGTTTCGGCCAGCTACAACCACTCATGACAAGTTTTGGAGTCAATCATGTGACCCACACACAGCAGATCTAAATCCGAACAAATTCGTTGAACTGGCTGAATATGACTGGACTACAACTGGCGCCTCTGGATCCACTGCAATAACATTGAACCTACCAAATTGCTTTTACCTTGATCGGGATTTTCCGGCTTTTGGACCAACAAGATATTTTGCTGCGATGAGATCCTCTTTCGACTTCCAAATACAGGCAAATGTTGCAACTGGATGTGCCGGCTCCCTGCTAGTGGTTTACTGGCCTCCCGGGGTCGTTACCACTGACAAGGAATTCAGGTCTTTCTTCAACAATCCCAATGTCATTCTCAATGTGGGCACATCAACAACAGCCAACTTACACATTCCATACACCAACTTTCGGAACTATGTGAGCACGGATACTACTGATCTTGGAACACTGAAGGTTTACGTCTTGGCCAATTTGGAAGTACCCACAGGGACACCAGGTAGTGCCACTGTTAACATCTATGGTGCGATGAGTCAACTTGACTTACAGTGCCCACGAGTTGTCCCTCGTGGACCAGCCCGTGAACGTGTTGATGTGGCCGAAGGAGTTGGAGCCATGAATCTGAGCAATTCAACAGTGACAAAAACGTCTAATTCCATGGCACTTGTGGGAGAAGATGTTGCCTCAGACCCCACCACAGCAGGCATGACAGGAGAAGTTGATGATTTATTGATTATTGCCAGAACACCCAACATCACATTATCAGCATCAACAGCCATCCAGAGTTGGAGTTCTACTGTGGCAAAAGGGACTTCTATTTTTTCCACTAATTGGTCGATTGCAACACTCAGCCCAAACATGCAAATGTTCTCAAATGGTTTCCGCTTTATGCGAGGCAGTGTTGTCATCAGGATAACAGTCTTCAACTCCACTCTCCACAAGGGTAGATTGAAGATAGCCTTCTATCCCTCTTCAGGGTCTGGACAGGCTTTTCCTGCACCATTCACAAACGCTGCAGCAAAGAACGCTTTCTTCGCCATTCTGGACATTGGACTCCAATCCTCCATTGACTTGACCATTCCGTTCACAAGCTTGTCTTGGGTCAAACAGATGACGGACAATTTTGGAAGACTCGAAATTTTCGTTCTCTCAAAGTTGTCAGTCACTCCTGCTACTTCACCAAGTGTCAAGTACATGGTCCAGGTCTTTGGAGGTGAAGACTTCCAAATGATGGCCCCAACCGATCGTGGCCTGATTTATCGCAGTCCCCCAGTGGAAAAAGAGGAAACCACTGTTGTCAACAACAGTGAAGAGGCAGCAGTCGCTGCTGGAATGAGGAGTCCTGAGAACACAAACGAGAAGGAGGAAGCTATGAATCCCACCGCCGTGATTGGAGTTCCAAGCATGTTGAATGCAAAGATTGAAAAAGTTCAAGTGACAAAAGCACCACATACCAAGATAACCAGCATTCTGGGGAGAGCACAATACCTTGGAAAATACACGGTCACTGGGACAACTGTTGTGCAAGTGGCACTGCCACTACCAAGCTCTGGATACATGTCTTTCTTGAACTTGTTCGCTTATTGGAGTGGTCCCATAACACTTCATATATTGAACCAAACCACTGAGGAAATGATGGTCTCCCACTCTTACTACGCAGAGACACCTGATGAGTCACTGATCACCACGGAAGGATGCATGGTTATCCCAGCTGGACAAAACGCATCCATGAAGTGCCCATTCTACTGGCACGAACCTCTGCGAAAAGTTTCTGAGGACCAAACATTCGGATTCATGTCCATGAAAACATTTGGAACAGGAGAAATACAAGTCTTCATCTCACTTGGAGAAATCAAACTCTACACGCCAGTTGCTGTCAAATTGATCTCGACAGCAAGAGATTGGATCACAAAACACCAATTTGACACTCTTGAACTCCCA

GGACAGAATGCAGTTGTGATGTATTTGAAGGATGGAAAACCTGACACACCATTGATGCCGAAAGAAGAACCACCACTACGACACAGGACACGGATCATGGATGCTTTGATTGGTAAAATTGTCAAAATTGACAAGGATAATGAGGTTGAGAACTCATGGATCAAGGACCTAACAACAGAAGGCATTGAGTCCAACCCTGGACCTGTGGTCCAATTGGTCTATCTGGATCGTGGCCTCTATAAACATTATGGAGTGGCACACGAAGGTTGGGTCTTGAATGTCAACTCAGACAACATTGCTGCAGCAGCAGCGACAGGAATGGTGTCAGTTCACTTGACAAAACAGACCCCAGAATGGATTGTTGATTCAGAACATGATATATCAGTCTTGAGAATGGCTGCATTGGAGAAGTCAGCACACACACCTGTTCGGTTTTCAGCAGCGGAAAATTGTGAAACTTGGGCCTACAAGGCACTTGGCATTGAGCGGATCACGCAAGCTAGAGCACTAGCTGTCTTTGGAATCATTGCTACTGTAACAACAGGAGCATTGGCACTTGCAAAGGGTGAACCGGTGAAAGCCATAAAGGAAGGTTTCACA

AAGGCTGGACAGTATGTTGGCAATGCATTCAGACATGGGGGTGGATGGGGGTTCATGAACTTCTTGAAAACAGAAACTTTTGACAGCATTAAGTGTGATATCATCAAGACACTTGCCAAGTTGATACTCCGGACTACGTGTTATGGGATCTTGTTCTGTAGCTGCCCAGGTTTGTTGACAGGAGCCGCAGTCATCAGTTTGATTGCAATGGATCTGACTGCTGTTGAAGGATTGTCAAGAAGTACAAAAGACCTGTTGACTGCATTGTTGGAAGGTGATTTGGCTAAGGCAGTGGATGCCATTTCAACTCTGACACTGGAGAATTCAGGAGACAGATGTGCTTTTGTGGCAGAGGCCAGTCGCCAACTGAACATCAGTTTATCTGGCCTTGAAGGGAAAAACACACCCCGAGGGTTGAAGGATTTCAACGAAGCAAGTCTTGGAGCGAGGAATGTTGAATACTGGATAGGAATGGCTAGGAGATTGTGGGCATTCATCAAGGACCTCTTTAATCCGGAGGAGAAGACACTTATCCAAGAATGGTTTGATGAAAGAACTGATGTGGTTGCACAAATCATGTACGATGCAGATCAAGTAATTTTGGAATCAGCAAAAGCTGAAGTCAGGAGGAACAAAACATTTCAGAAACATACCATGCAAGTCGCGGAACGACTTTGTCGCCTTAAAACGTTAGCAATGAAAGGAGGCATGTTCCAGATCGTGGCTACATGTACGAACATGATAAACAAACTCTCAAACATCCCAAAACCGCGACCACAATCAGGAAACATATTTCGCATGGAACCAATTGGCATCTGGATTTCAGGCCCAGCAGGATGTGGGAAGTCACTAATCACCACAGAAATAATGTCACAATTATTGGATTTGATACAAACAGACAATGAACTCTGCCAGAAATTGGACGCAGAGAATCAGGAACCCAGTACGGTCTTCACCCACCCAACAGGAGCGGAACACATGGATGGATACCAAGGACAGTTTTTCCACATCTTAGATGACATGGGCCAAGCACGTGAGGAGATGGACATGAAATTGCTCTGCCAAACCATTTCTTCAGTTCAGTACTCACCACCAATGGCAGATCTCCCAGATAAAGGTAATCGATACACCAGTAAGATAGTTATAGCATCAACAAACAGAACTGACTTCGACACCGTTGTTTTGACAGACCCCAAGGCGCTTGAGAGGAGATTCGCATACAAATTGCGAGTCAAGTGTGCTGATGCCTTCACAAGTGGA

GTTACAGGGAAGATTGACACTGATTTGGCTATGAAGAAAGGTGCCATATCGACTGGTGCGGCATGGATGCTTGGAGATGGAAATGGCTTCAGACAAAAGGAAACACTTCCCATGCAAAAGATCGTCAGGAGCATTTTTGAGGAATTCAAAACAAAGATGGCAGTGTCAGAACGCATCAACTCGACACTACAGAAATTCAATGATGGAACCGTTGGCAAATTTGAACCCAAGCCATTGGTCAAATCAGTTGATCTGGTTAAGCGAAGTCTGGAAAATGTGCTCAAGCCCAAAGGACGTAAGCACATCAATCCTTTTGTCTATGATGGAACTGATGACATCGAAGAGAGCAGCAGTGATGAGGAACAAACCATTGAAGAACCTAAGGGGCAGGCATCCTTGGATGCTGCTTCTGAATATATGAGAAAAGCTGAAGTGCGATTAGAGAACATATTTCAGGACATTGTTGACAATGAAGATGGTTGGACACATGTGCGTTCATATGCTGCCACTCTTGTGGACTTGCAAAAGGAATGGGAGAAGACTTCTGCAAAAGAACAGATTGACGCACTGGACTGGAGGATGGATGTGCCAGATGCACCA

TTTGACTCACTGAAGCTACGGTTTGAAGGAAAAGCAGAAATGACAGCACCTGAGAAAATTAAGAACTGGATAAAAGAGAAAACCCTGAAAGCAAAGAATTGGCTAAAGAGATGGGGCCCATTGTTTTTGGTGGCAGGGAGCATTTTGACTATCATTGGCATTGTTGAAACGGTGGGATGTCTCACCAAGACATGGACCGTTGAAGCGGCTTTGAACAACATGGATGGAAAGATACAAAAGAATTTGTCGTCAATGGAGAAAGCAATGGACGCCCTTGAAGAACTAACAACCCCTTTGGAGAAAACAACTGAGAAAAGACCTTACAGTGGTAATCAACAACCACAGAAGAAAGTTAAAGTGAAGAAAGAAGTGTACCAGCAGAAAGGAAGACACCCAAGCCCACAAGAATATGCACACTTGGCTAAATTTTGTGTGTCAATGAGAGATCAGGATAATCAACATGTCTATGGCATGACTTCTGGACTAGCACAAGTGATCACCTACAAGCATTTCTTTGAGACAGTTGGAGGAGGCACAGTCAAGAAATTGAACTGGAATGGATTGACAGCAGACATTGAGGACTATGAGTTGACATACTTGGAGAAGGAAATGCCTGATGGCAAAGTCCTTGAGACGGATCTCGTCAAGGTTGATTTTAAGAACTTACCTTTCCGCATGCCCAACGCCAGCAAACACATTACTGATGACTTTGAAGAAGGAACTGAAGGGGTTCTGTTGTATGGGGGCTTTCTTGGGTGCTTTACCATGAAAATGGGACCAGTCAAGAGGGAGGCCATGTACACAGTTATCCAACAGGAAGCCTACAGTACCATGTACTTTGATGGTTACAGATACCCAGGAGAGACGGAATTGGGTAGCTGTGGTGGTTTGCTGATTGCCAAAAGCAAAGGAAGTTGGAAGATTCTTGGAATGCACCACGCAGGGAATGAGACAAGTAGTTTGGCTACGAAGTTGGACCCAGTGATGATGGCTAAAGGTGTGGTCATTGACAAGAAACCTGCACCAAGTCCAAGTCATCTATCAAACAAGACCAAACTGAGAAGGAGCCCACTGTACGGGATAGTACCAACAAAAATGGGACCAGCGCCATTGACACAAAATGATACCAGGATTGACCCAGAAAAGAAACCAGAAAGTTTGATCAAGAAAGCCTGTGATAAATACAGAACCGATAAATTTGAACCAAACAAGGAACACTACACCAAGGCACTGAACTACACTACCAAGACCGTTTTTGCAGCAGTTGGACGCATACAAAATATGTGGACAATCGAAGAAGCGATCACAGGACAGGGAATGAATCCGATTGATATGAAAACATCACCAGGGGACAAATATGTGAAAATGGGTCTGAAGAAAAGTGATCTGATCATCTGTGATGGGAAAGGAGGTATGATCCCCTCACAAAAACTGAGAGAAGACGTTGAAACCATGATAGCAAGATTGAACGAAGGACAGGAAATCAACACAATGTTCAAGGCCACCATCAAGGATGAAATAATCAAGAATGAGAAAATCGAAATTGGCAAATCAAGATGCATTGAAGCTTGTGAATTGGATTATGTTGTAGTCTACAGGATGATCATGGGACCAGTCTATCAGAAAATTTATTCAACATCTGCATTGAGGACTGGAGTGGCAGTTGGCATCAATTGCTACACTGACTATCATGAACTGGCCATGGCGATGACTGGTGACATGTACGCCCTGGACTATGGAACTTACGATGGGTCACTACCAAAACAACTGATGGAGGACGCTGTTGGTGTGCTCGCATCTTGCACCCTGGACCCAGAAATGGTCATGGCACTCCACAAGCCAGTGGTGGTATCAGAGCATCTGGTCTTGGATGAAATTTGGACAGTTCAGGGTGGCATGCCCTCAGGTTCTCCATGTACAAGTGTTCTCAACAGCATTTGTAACCTCATTGTGTGTCGCACAGCTTTCGCACAAGCAGGACTTGAAGACATGAGCACAATGGCCATTGTGACATACGGGGATGATGTTTTGGCAAGCATCAATCAAAATGAACCAATCCGAGATGAGGAAATCCCAGAAATAATTAAAGCATCATTTGGCATGGATGCTACATCCGCAGACAAAACATCAAGCAATCTGCGGGTTGACTTTGAGGAAGCAACCTTTCTCAAGAGAAGATTCCGACACTTCCCAGGAACAAGATTCATCGTCGGTCAATTGGACCTGGCATCAATGTTGCAAAAAATACAATGGTGCCATGGAAAGGAAGAATTCAAGCAACAGATTGAAAGTTTTTGCTTGGAACTCGTTTTGCACGGAGAAGTTGCATACGAGAAGGTCAGAAAGGCAGCCCAACCTGCTCTCGACCGTTTTGGTGTGCTAATTACACCATATTCGCTCAAACATGCGGAGTTTTATTACAAAATGTTTGAA**TAA**GAGGTATAGAGGTAACTTTAGGTAGCCTAGTAAATCTGTAGAACCCGTCAGGAGTTATGTCCAGGTAATGCAGTATAGTGTGCCTACTGTTGATCTTAGTGGTAATTGAGTTAATATCACCAATCTATATAAAAACACAAAAAACTTT
